# Acute respiratory distress secondary to posterior mediastinal goiter: a case report

**DOI:** 10.1186/1757-1626-2-7458

**Published:** 2009-05-18

**Authors:** Dawn E Jaroszewski, Faisal G Bakaeen, Joseph Huh

**Affiliations:** 1 Department of Cardiothoracic Surgery, Mayo ClinicPhoenix, ArizonaUnited States; 2 Department of Cardiothoracic Surgery, Michael E. DeBakey Veterans Affairs Medical Center HoustonTexasUnited States

## Abstract

Large posterior mediastinal goiters are extremely rare. Progressive enlargement and possible compression of adjacent structures, as well as malignant potential necessitate that these goiters should be surgically excised. A review of mediastinal tumors, specifically intra-thoracic goiters is presented along with a case report of acute respiratory compromise secondary to tracheal compression by a large posterior goiter.

## Case presentation

**Figure 1.**
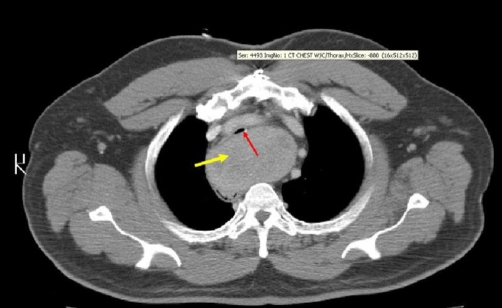
Computed Tomography of the chest showing a 9 cm posterior mediastinal mass (yellow arrow) compromising the tracheal lumen (red arrow).

A 59-year-old African-American man with past medical history significant for cervical thyroidectomy for goiter five years prior presented to the emergency center with acute strider and respiratory embarrassment. His oxygen saturations remained stable at 99% on a 50% non-rebreather face mask with a respiratory rate of thirty-two breaths per minute. The patient had been seen in consultation by the thoracic surgical service 6 months prior with the complaint of dysphagia and chest discomfort. He was found on computer tomography (CT) scan to have a large 9 cm posterior mediastinal mass adjacent to the trachea and displacing the esophagus. Serum markers were non-diagnostic. He was recommended for biopsy and surgical removal however he failed to return for any further surgical or medical intervention until his current presentation. The patient reported a recent upper respiratory tract infection including productive yellow cough, runny nose and subjective fevers. He had not sought any treatment until with continuous coughing overnight he experienced progressive stridor and respiratory distress which led him to his presentation to the emergency room. A repeat CT scan revealed the mass encroaching and compromising the tracheal lumen with displacement of the esophagus laterally (Figure 1).

**Figure 2.**
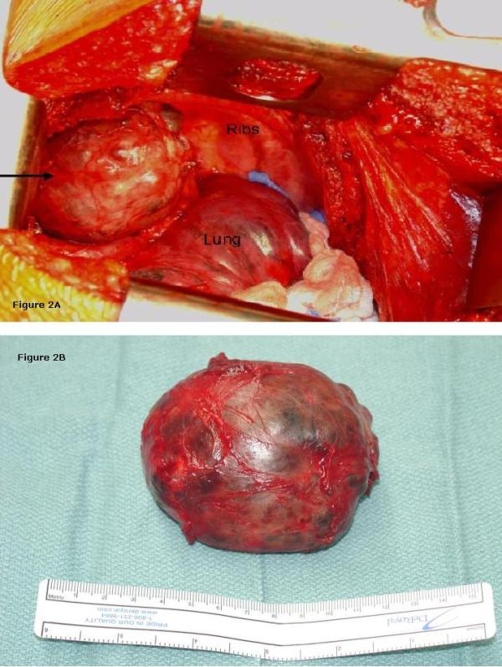
**(a)** Intra-operative photograph showing right posterior mediastinum. The black arrow points to a large goiter. The lung is compressed and held down with sterile gauze. **(b)** Gross pathologic specimen of goiter.

The patient was taken emergently to the operating room. Intubation was performed in the presence of the surgical team under direct fiberoptic visualization while maintaining spontaneous respiratory effort until the airway could be secured. A single lumen #6 endotracheal tube was passed beyond the tumor and positioned in the left main bronchus for single lung ventilation. A right posterior-lateral thoracotomy was performed through the fifth intercostal space giving good exposure of the mass. Gross distortion of the peri-tracheal structures was present secondary to the large space-occupying lesion (Figure 2a). The mass was densely adhered to the posterior mediastinum and right lateral tracheal wall, requiring meticulous dissection off these structures. After gross resection of the mass, a single chest tube was placed and the thoracotomy closed in a standard layered fashion. Final pathology was consistent with benign thyroid goiter (Figure 2b). Due to concerns of tracheomalasia, the patient was kept intubated 48 hours following surgery. After endoscopic confirmation of airway patency, the endotracheal tube was removed under direct visualization. The patient did well, however, was noted to have persistent hoarseness and vocal cord paralysis consistent with right recurrent nerve injury. Surgical medialization of his vocal cord was performed and at 6-month follow-up he was doing well.

## Discussion

Many types of tumors and cysts occur in the mediastinum (Table 1). These are more frequently diagnosed in young children and middle-aged adults [1]. More than half are discovered incidentally on a routine radiographic examination of an asymptomatic person. When symptomatic, the presentation is usually due to the size, location, and compression or invasion of mediastinal structures. Cough, dyspnea, chest pain, and dysphagia are the more common presenting symptoms [2]. In the anterior mediastinum, thymoma, germ cell tumors, lymphoma and aberrant thyroid tissue most frequently occur. The posterior mediastinum more commonly has tumors of neurogenic origin as well as lymphoma [1].

**Table 1.** Common mediastinal tumors

Anterior Mediastinum	Middle Mediastinum	Posterior Mediastinum
Thymoma (most common tumor in adults)	Lymphoma	Neurogenic tumors
Germ Cell Tumors	Bronchogenic Cysts	Cysts
Intra thoracic goiter	Pleuropericardial cysts	Schwannomas
Lymphoma (most common tumor in children)		Ganglioneuromas
Parathyroid Adenoma		Neuroblastoma
Teratoma		Neurofibroma

The diagnostic approach to patients who have mediastinal masses should include thorough imaging. CT is the imaging modality of choice for evaluating a suspected mediastinal mass and can provide useful information for surgical planning [3,4]. Diagnostic evaluation may include serum and urine biochemical marker and interventional biopsy if the mass is safely accessible [4]. Thyroid scans can also be performed however they often fail to show the intrathoracic goiter [5].

Intrathoracic ectopic goiters are rare, but have to be considered as a diagnostic possibility in all mediastinal masses. The displacement of the thyroid tissue inferiorly in connection with the embryogenesis of the heart and the large vessels explains the etiology of the disease [6]. Intrathoracic goiters are classified into two groups according to their location in the anterior or posterior mediastinum. The majority are located retrosternal in the superior and anterior mediastinum. Both retrotracheal and retroesophageal posterior mediastinal locations are extremely rare [1,2,4,7,9]. The presenting symptoms generally relate to the compressive nature of the mass on nearby structures. In a review of 52 cases by Sanders, half of the patients were asymptomatic at presentation. The other half of the patients had at least one compressive symptom [2].

Once the diagnosis of an intrathoracic goiter is obtained, the treatment is surgical. The propensity to enlarge and compress adjacent structures as well as the small chance of malignancy necessitates resection [2,4,7,8]. Although many goiters remain clinically silent, they can produce sudden and unpredictable respiratory distress [9]. A recent respiratory infection with increased secretions likely exacerbated acute near collapse of the airway in our patient. A small percentage of goiters will also show signs of neoplastic degeneration (3-17%) [2,10,11]. Seventeen percent of the intra-thoracic thyroids in a case series by Sanders showed malignancy, 21% included incidental papillary carcinomas [2]. None of these were identified pre-operatively by length of goiter presence, symptoms, or fine-needle aspiration.

The only effective treatment for mediastinal goiter is surgical excision. Perioperative management should include careful evaluation of the airway as the extent of compression and deviation caused by the mass can lead to a difficult intubation. A multidisciplinary team approach is critical and includes the surgeon, anesthesiologist, and endocrinologist. Anesthesia for patients with mediastinal masses carries a significant risk for fatal or near-fatal cardiorespiratory events [5]. Careful history taking and thorough preoperative investigation and imaging identify the patients at greatest risk. Careful planning including securing the airway at the time of surgery is key in the management of such patients [3,5,7]. Preoperative fiberoptic bronchoscopy performed by or involving the anesthesiologist is invaluable for determining the plan for intubation and ventilation.

Both cervico-sternotomy and thoracotomy approaches have been described for excision [2-4,7-12]. Most anterior substernal goiters and some ipsilateral posterior mediastinal goiters can be removed safely through a cervical incision [2]. Large posterior mediastinal goiters, contralateral retrotracheal or retroesophageal posterior mediastinal goiters, and isolated mediastinal goiters with no significant cervical connection can be removed through a combined cervical and thoracic approach [2,11,12]. Right posterior-lateral thoracotomy was utilized in our patient due to previous neck incision and tumor location. Thoracotomy allowed excellent, safe exposure for removal of the goiter.

## Conclusion

Goiter is an occasional finding with intrathoracic masses. These are most commonly anterior however a rare posterior mediastinal thyroid may be found. Malignant potential and a tendency toward enlargement and airway compromise necessitates treatment. Surgical excision is recommended to treat symptoms and avert complications related to their mass effect.

## Abbreviations

CT: Computer Tomography
